# Role of myofibroblasts and collagen type IV in patients of IgA nephropathy as markers of renal dysfunction

**DOI:** 10.4103/0971-4065.62098

**Published:** 2010

**Authors:** R. W. Minz, A. Bakshi, S. Chhabra, K. Joshi, V. Sakhuja

**Affiliations:** Department of Immunopathology, Postgraduate Institute of Medical Education and Research, Chandigarh, India; 1 Department of Histopathology, Postgraduate Institute of Medical Education and Research, Chandigarh, India; 2 Department of Nephrology, Postgraduate Institute of Medical Education and Research, Chandigarh, India

**Keywords:** Collagen type IV, IgA nephropathy, myofibroblasts

## Abstract

The aim was to evaluate the role of a-smooth muscle actin (SMA) and collagen type IV as markers of chronicity in renal biopsies of IgA nephropathy patients and to correlate the degree of their interstitial expression with renal function as judged by serum creatinine. Renal biopsies from 29 clinically, histologically and immunologically confirmed cases of IgA nephropathy were reviewed to assess activity and chronicity indices. Immunohistochemical stains for α-SMA and collagen type IV was performed on 23 patients with adequate tissue available in the block. The interstitial expression of α-SMA and collagen type IV was then correlated with chronicity and activity indices, serum creatinine and 24 hours urinary protein. Pearson's coefficient of correlation, unpaired-t test were used for statistical analysis. α-SMA and collagen type IV were shown to be expressed in the interstitium in all 22 cases showing interstitital fibrosis. Both showed a similar distribution pattern with predominant periglomerular and peritubular positivity. The cases were divided into two groups (low and high grade) depending on the percentage of interstitial area showing positivity for these two antibodies. On statistical analysis, the expression of both a-smooth muscle actin and collagen type IV showed a striking correlation with the histological chronicity index (*P*<0.01). A positive correlation was also noted with the serum creatinine at the time of diagnosis. It is seen that an immunohistochemical approach to grading interstitial fibrosis as in this study is far simpler than the histological grading systems prevalent and is an important baseline prognostic indicator.

## Introduction

The natural history of IgA nephropathy has been extensively studied worldwide and it has been shown to follow a variable course with up to 30-35% of cases progressing to end stage renal disease.[1] Hence, there is a need to prognosticate renal function in all patients diagnosed as IgA nephropathy at presentation. Over the years, various clinical and histological parameters have been evaluated for this purpose.[2–7] It has increasingly been realized that the long term renal outcome correlates more closely with the tubulointerstitial changes rather than with the severity of glomerular pathology.[8] This has paved the way for research into the mechanisms underlying interstitial fibrosis in order to identify early and potentially reversible stages in the progression of renal disease.[8–10]

Myofibroblasts have a pivotal role as mediators of interstitial fibrosis and glomerulosclerosis, which can be used as a prognostic marker in renal disease. They express a wide range of cytoskeletal proteins, in particular, a-smooth muscle actin (α-SMA) and to a lesser extent vimentin and desmin. Although the exact origin of these myofibroblasts remains uncertain, emerging evidence indicates that mature tubular epithelial cells are capable of transforming into myofibroblasts under pathological conditions, a process that is called epithelial-mesenchymal transition. Several cytokines and growth factors regulate this epithelial-mesenchyme transition.[11] The role of myofibroblasts in the pathogenesis of renal fibrosis has been reported in many clinical nephropathies including IgA nephropathy.[12–16] Collagen type IV, though normally present in the basement membranes has been shown to appear in the interstitium during interstitial fibrosis in a similar distribution as myofibroblasts.[15,17]

This study was undertaken to assess the significance of α-SMA and collagen type IV as markers of chronicity and/or activity in renal biopsies of patients with IgA nephropathy. An attempt was also made to correlate the degree of α-SMA and collagen type IV expression with the renal function as judged by serum creatinine levels.

## Materials and Methods

All adult cases diagnosed to have 2+ or more than 2+ dominant/codominant IgA deposits, predominantly in the mesangium, over a five-year period from January 1995 to January 2000 were studied retrospectively. The diagnosis of IgA nephropathy was established in 32 cases by a combination of clinical, histological and immunofluorescence findings. Antinuclear antibody testing and liver function tests were performed in all the cases to exclude secondary causes of IgA nephropathy. Cases of Henoch Schonlein purpura and SLE showing IgA dominant pattern were excluded from the analysis. Clinical parameters including serum creatinine and 24-hour urinary protein were recorded in all patients at the time of presentation. Three microns thick paraffin sections were cut and stained with haematoxylin and eosin (H and E) and periodic acid Schiff (PAS). Out of these 32 cases of IgA nephropathy, three cases could not be evaluated histologically to assess chronicity and activity scores due to inadequate tissue available for light microscopy. So, renal biopsies from the remaining 29 cases of IgA nephropathy were reviewed with respect to the changes seen in glomeruli, interstitium, tubules and vessels by an experienced nephropathologist.

The biopsies were given a chronicity score based on the grading proposed by Ka Fai To *et al*.[6] Only those biopsies which included at least three glomeruli were taken up for this evaluation. The grading was done as under:

1. Glomerular grade (GG): The mean percentage of sclerosis per glomerulus was given a score ranging from GG 0 to GG 3.
2. Tubulointerstitial grade (TIG): The percentage area of tubular atrophy and interstitial fibrosis in renal cortex was scored from TIG 1 to TIG 3 regardless of inflammatory cells.
3. Hyaline arteriosclerosis (HA): If hyaline arteriosclerosis was absent, a score of 0 was given and if it was present, a score of 1 was given.

All three scores were added up to give a chronicity score with a minimum of 1 and a maximum of 7.

The biopsies were also evaluated to assess an activity index based on the scoring proposed by Wyatt *et al*.[7] The following parameters were scored:

1. Glomerular proliferation was scored from 0-3.
2. Interstitial inflammation was scored from 0-3.
3. Crescent formation was scored according to the percentage of glomeruli showing crescents on a scale of 0-3.

All three scores were added up to give an activity score with a minimum score of 0 and maximum of 9.

The chronicity and activity scores were then correlated with serum creatinine and 24 hours urinary protein as well as with each other using the Pearson's coefficient of correlation.

### Immunohistochemical analysis

Immunohistochemical stain for α-smooth muscle actin and collagen type IV was performed on 23 cases where adequate tissue was available in the block. Avidin biotin peroxidase method was followed. In order to define favorable and unfavorable prognostic groups, positive interstitial staining for α-SMA and collagen type IV was graded into low and high grade based on the percentage of interstitial area showing positivity:

≤25% of the interstitial area showing positivity: Low grade (favorable)

>25% of the interstitial area showing positivity: High grade (unfavorable).

Both low and high grade groups were compared with respect to mean chronicity and activity scores as well as with mean serum creatinine and 24 hours urinary protein values using the unpaired-t test to look for any statistical significance. Glomerular staining for α-SMA and collagen type IV was also assessed. Significant glomerular staining was noted only with collagen type IV.

Positivity only within capillary basement membrane was recorded as normal, focal weak positivity in the glomerular mesangium as low and diffuse strong positivity in the glomerular mesangium as high.

## Results

A total of 32 adult cases (4.5% of all biopsied cases) of IgA nephropathy were diagnosed over a five-year study period whose clinical and pathological spectrum has already been reported in a recent paper.[18] It was seen that 29/32 renal biopsies that were re-examined histologically to assess chronicity and activity scores showed the full range from 1-7 and 0-9 respectively. Twenty eight cases showed a correlation between their chronicity and activity scores; one case showed a high activity score and a low chronicity score. This case had an unusual histology of severe tubulointerstitial nephritis with glomeruli showing diffuse mesangial proliferation. This case had presented clinically with gross hematuria and oliguria progressing to anuria and thus represented a dual pathology of IgA nephropathy with possible drug induced tubulointerstitial nephritis.

The chronicity and activity scores were correlated with serum creatinine and 24 hour urinary protein using the Pearson coefficient of correlation. While serum creatinine showed a significant correlation (*P*<0.01) with both chronicity index and activity index, 24 hours urinary protein slowed no such correlation.

An attempt was also made to define the cut off value for activity score and chronicity score, which could divide the cases into good and bad prognostic groups based on the serum creatinine levels. A cut off value of four for activity index was proposed and the results are shown in Table 1. The cut off for chronicity index was taken as three and the two groups (total 28 cases) were compared similarly as shown in Table 2. The above mentioned case was excluded from this analysis due to the dual pathology it showed. The severe tubulointerstitial nephritis accounted for the high serum creatinine (9 mg/dl) in spite of a low chronicity score.

**Table 1:** Correlation of activity index with serum creatinine (n = 29)

**Activity index**	**No. of cases**	**Serum creatinine**
≤4	17	1.74±1.09
>4	12	4.26±2.82
*P* value	-	<0.01

**Table 2:** Correlation of chronicity index with serum creatinine (n = 28)

**Chronicity index**	**No. of cases**	**Serum creatinine**
≤3	13	1.26±0.55
>3	15	3.69±2.18
*P* value	-	<0.01

Immunostaining for α-SMA and collagen type IV could be performed in 23 cases in which adequate tissue was available. In one case α-SMA could not be interpreted due to inadequate cortical area while in another case collagen type IV was not interpretable due to poor stain. In all, immunohistochemical analysis was available in 22 cases.

### α-SMA

**Figure 1:**
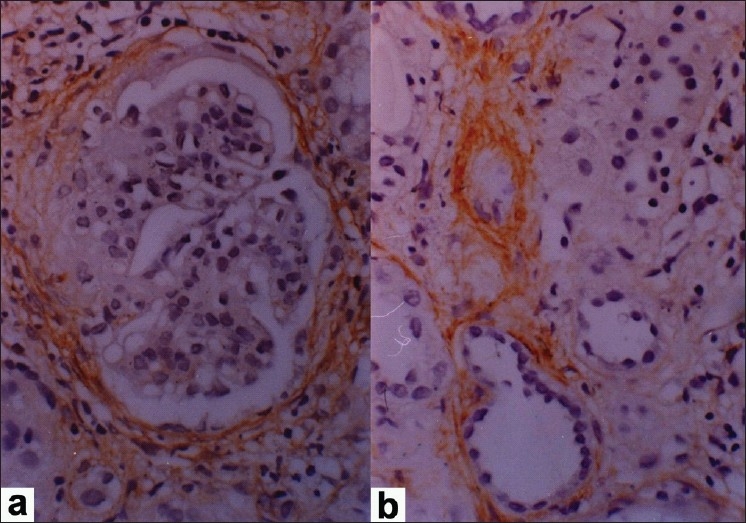
Photomicrograph showing (a) periglomerular α-SMA positivity and (b) peritubular α-SMA positivity (IP,×132OM)

**Figure 2:**
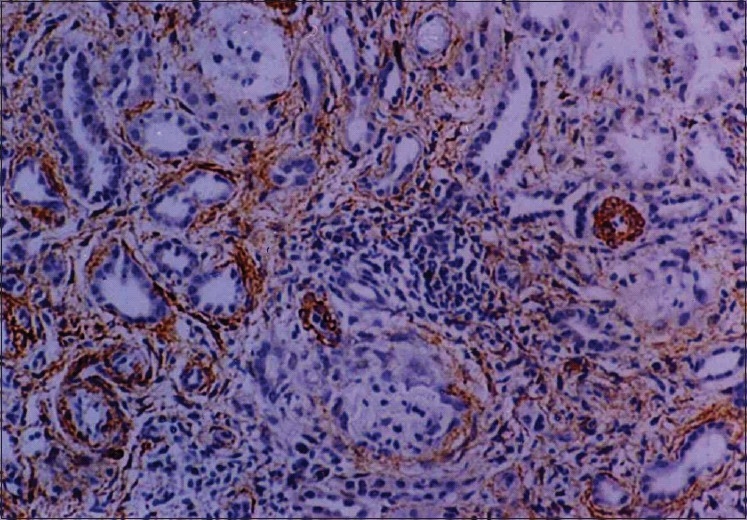
Photomicrograph showing high grade diffuse interstitial positivity of α-SMA (IP,×66OM)

All 22 cases showed an internal control of strong positivity in the vascular smooth muscle. Variable staining for α-SMA was detected in the interstitium ranging from few sparse cells to strong positivity covering more than 50% of the biopsy area. The α-SMA positivity appeared maximally in periglomerular areas followed by peri-tubular and perivascular areas from where it appeared to radiate into the surrounding interstitium [Figure 1]. Low grade α-SMA positivity was seen in nine cases while high grade positivity was noted in 13 cases. In the high grade cases, a fine meshwork of fibre-like positivity was noted in the interstitium and scarred areas [Figure 2]. Interstitial α-SMA significantly correlated with both chronicity index and activity index with a stronger correlation with chronicity index (*P*<0.01). A statistically significant correlation was noted with serum creatinine, but not with 24 hour urinary protein [Table 3]. No significant staining for SMA was noted within the glomerular mesangium or crescents or sclerosed segments.

**Table 3:** Correlation of interstitial α-SMA with chronicity index, activity index and serum creatinine (n = 22)

α**-SMA**	**No. of cases**	**Chronicity index**	**Activity index**	**Serum creatinine**
Low grade	9	2.44±2.00	2.78±2.81	1.61±1.89
High grade	13	4.85±1.46	4.92±1.49	3.69±2.13
*P* value	-	<0.01	<0.05	<0.05

### Collagen IV

**Figure 3:**
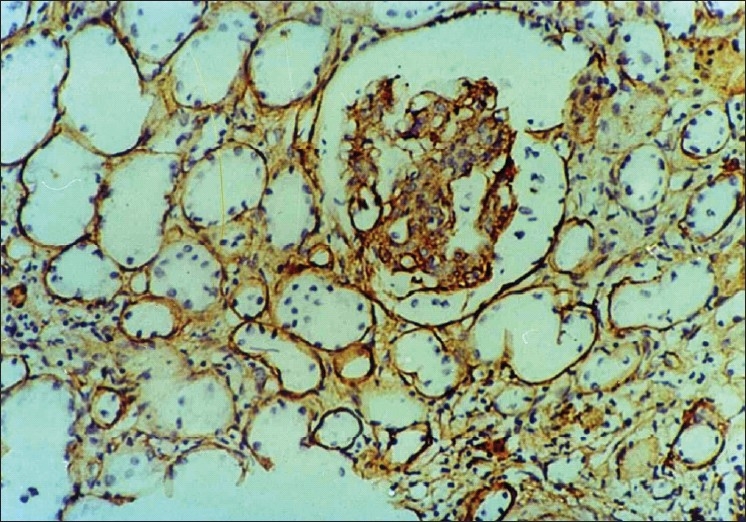
Photomicrograph showing low grade interstitial positivity of collagen type IV (IP,×66 OM)

**Figure 4:**
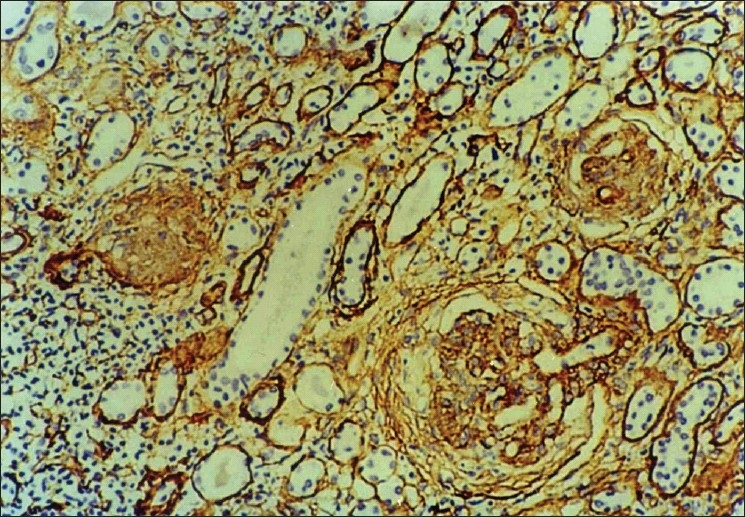
Photomicrograph showing high grade interstitial positivity of collagen type IV with prominent periglomerular distribution (IP,×66 OM)

Collagen IV was present normally in the tubular basement membrane, and glomerular capillary loops. Due to variable fixation of the kidney biopsies, the staining intensity of collagen IV varied from case to case with minimal or no staining in poorly fixed areas. Interstitial collagen IV followed a similar distribution as α-SMA and was found in periglomerular areas and also in scarred areas. Tubular atrophy in the scarred areas was also well highlighted by collagen IV. Low grade collagen IV positivity [Figure 3] was seen in nine cases while high grade positivity [Figure 4] was noted in 13 cases. As shown in Table 4, interstitital collagen IV showed good correlation with chronicity and activity index as well as with serum creatinine, but not with 24- hour urinary protein.

**Table 4:** Correlation of interstitial collagen IV with chronicity index, activity index, serum creatinine and 24 hr urinary protein (n = 22)

**Collagen IV**	**No. of cases**	**Chronicity index**	**Activity index**	**Serum creatinine**
Low grade	9	1.78±1.09	2.67±2.44	1.13±0.57
High grade	13	5.46±1.12	4.85±1.77	3.78±2.31
*P* value	-	<0.01	<0.05	<0.01

Glomerular collagen IV staining was also detected ranging from normal to high grade. Five cases showed normal glomerular staining for collagen IV while nine cases each had low and high grade staining. Staining was noted in areas of mesangial expansion and also in fibrous crescents; however, globally sclerosed glomeruli did not reveal significant collagen IV staining. On statistical analysis, glomerular staining for collagen type IV did not show significant correlation with either chronicity and activity indices or with creatinine/24 hour urinary protein.

## Discussion

Although there is extensive literature from the West on epidemiology, clinical features, pathological findings and prognostic factors of IgA nephropathy; studies from India have been limited.[18–23] IgA nephropathy is the most common glomerunephritis worldwide and up to 30% patients progress to end stage renal disease. Thus, it is imperative to identify factors allowing not only prediction of renal outcome but also the detection of high risk patients at the time of presentation, which is important in planning the long term management of these patients.

Though a wide range of glomerular histology is noted in IgA nephropathy, this has not been found to be predictive of long term renal outcome. Increasingly, the importance of tubulointerstitial changes and glomerulosclerosis has been emphasized in the progression of renal damage.[3,8] Hence there has been an increasing interest in the past decade in the pathogenesis and in the search for early markers of tubulointerstitital changes in order to prognosticate renal functions.

Based on such studies, we evaluated the tubulointerstitial and glomerular changes of 29 cases of IgA nephropathy by calculating the chronicity and activity index based on the criteria proposed by To *et al.*[6] and Wyatt *et al*.[7] We proposed a cut off of 3 for chronicity score and 4 for activity score for dividing patients into good and bad prognostic groups. In the bad prognostic group, most cases showed both high chronicity and high activity index. Both indices showed a significant correlation with the serum creatinine. Neither the chronicity nor the activity index showed any statistical significant correlation with 24-hour urinary protein levels. Thus, in this study, baseline proteinuria at the onset does not appear to predict the prognostic outcome. Coppo and D'Amico also showed the relevance of proteinuria during follow-up more than proteinuria at onset thereby emphasizing that follow-up studies are needed to substantiate the value of proteinuria as a marker of progression of IgA nephropathy.[24]

In spite of the usefulness of chronicity and activity indices in prognosticating renal function, these are cumbersome, so there is a need for markers of tubulointerstitital changes which are simple, reproducible and can accurately predict renal function. Our results on the immunostaining for α-SMA and collagen type IV showed significant correlation with both chronicity as well as activity indices with both showing a greater statistical correlation with the chronicity index (*P*<0.01). Thus α-SMA and collagen type IV can be used as markers of chronic histological lesions in renal disease. Similar observations were made by previous authors on IgA nephropathy where they found interstitial myofibroblasts to correlate both in degree as well as distribution with interstitial fibrosis.[14,15] These authors, in addition, correlated interstitial myofibroblast expression with the long term renal outcome as judged by the change in serum creatinine values over the observation periods. They found interstitial myofibroblasts expression to be an independent and superior prognostic marker of progressive disease compared to the best conventional histological predictive parameters namely tubular atrophy and interstitial fibrosis.

Goumenos *et al*. showed that interstitial collagen type IV also correlates with the long term renal outcome.[15] Our study was limited by its lack of follow-up of the cases; therefore we cannot directly confirm or refute these observations. However, we correlated interstitial α-SMA and collagen type IV staining with the serum creatinine values at the time of presentation and found a positive correlation. Interestingly, there were two cases where in spite of low chronicity and activity index, a strong staining for interstitial α-SMA was noted. We predict that these were the cases that would have had a more progressive course had a follow up been available.

Thus this study, though small, has highlighted an important fact that immunohistochemistry for α-smooth muscle actin and type IV collagen provides an easier method of grading interstitial fibrosis (in comparison with existing cumbersome histologic grading methods) into good and bad prognostic groups. In future studies, it is suggested that immunostaining with a-smooth muscle actin and collagen type IV can be quantified more accurately using automated digital imaging making assessment of tubulointerstitial fibrosis much more simpler and reproducible for routine use. The above findings can be analyzed in larger cohort studies with adequate follow-up.
